# Effectiveness of cardiac rehabilitation programs on medication adherence in patients with cardiovascular disease: A systematic review and meta-analysis^☆^

**DOI:** 10.1016/j.ijcrp.2023.200229

**Published:** 2023-12-12

**Authors:** Lemlem Gebremedhin Gebremichael, Stephanie Champion, Katie Nesbitt, Vincent Pearson, Norma B. Bulamu, Hila A. Dafny, Shelda Sajeev, Maria Alejandra Pinero de Plaza, Joyce S. Ramos, Orathai Suebkinorn, Aarti Gulyani, Lemma N. Bulto, Alline Beleigoli, Jeroen M. Hendriks, Sonia Hines, Robyn A. Clark, On behalf of the NHMRC CHAP Project Team

**Affiliations:** a Caring Futures Institute, College of Nursing and Health Sciences, Flinders University, Bedford Park, SA, Australia; b Mparntwe Centre for Evidence in Health, Flinders University: A JBI Centre of Excellence, Australia; c JBI, School of Public Health, The University of Adelaide, Australia; d Flinders Health and Medical Research Institute, College of Medicine and Public Health, Flinders University, Bedford Park, SA, Australia; e Centre for Artificial Intelligence Research and Optimisation (AIRO), Torrens University, Adelaide, South Australia, Australia; f College of Science and Engineering, Flinders University, Adelaide, SA, Australia; g National Health and Medical Research Council, Transdisciplinary Centre of Research Excellence in Frailty and Healthy Ageing, Adelaide, SA, Australia; h Centre for Heart Rhythm Disorders, University of Adelaide and the Royal Adelaide Hospital, Adelaide, SA, Australia; i Flinders Rural and Remote Health, NT. College of Medicine and Public Health, Flinders University, Australia

**Keywords:** Cardiac rehabilitation, Clinical outcomes, Effectiveness, Medication adherence, Standard care, Systematic review

## Abstract

**Background:**

Education to improve medication adherence is one of the core components of cardiac rehabilitation (CR) programs. However, the evidence on the effectiveness of CR programs on medication adherence is conflicting. Therefore, we aimed to summarize the effectiveness of CR programs versus standard care on medication adherence in patients with cardiovascular disease.

**Methods:**

A systematic review and meta-analysis was conducted. Seven databases and clinical trial registries were searched for published and unpublished articles from database inception to 09 Feb 2022. Only randomised controlled trials and quasi-experimental studies were included. Two independent reviewers conducted the screening, extraction, and appraisal. The JBI methodology for effectiveness reviews and PRISMA 2020 guidelines were followed. A statistical meta-analysis of included studies was pooled using RevMan version 5.4.1.

**Results:**

In total 33 studies were included with 16,677 participants. CR programs increased medication adherence by 14 % (RR = 1.14; 95 % CI: 1.07 to 1.22; p = 0.0002) with low degree of evidence certainty. CR also lowered the risk of dying by 17 % (RR = 0.83; 95 % CI: 0.69 to 1.00; p = 0.05); primary care and emergency department visit by mean difference of 0.19 (SMD = −0.19; 95 % CI: −0.30 to −0.08; p = 0.0008); and improved quality of life by 0.93 (SMD = 0.93; 95 % CI: 0.38 to 1.49; p = 0.0010). But no significant difference was observed in lipid profiles, except with total cholesterol (SMD = −0.26; 95 % CI: −0.44 to −0.07; p = 0.006) and blood pressure levels.

**Conclusions:**

CR improves medication adherence with a low degree of evidence certainty and non-significant changes in lipid and blood pressure levels. This result requires further investigation.

## Graphical abstract

**Figure:**
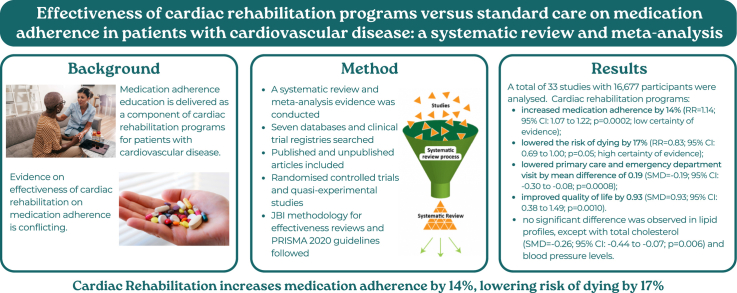

## 1 Introduction

Cardiovascular disease (CVD) is the leading cause of death worldwide, taking the lives of around 17.9 million people every year [1]. Heart attack and stroke are the most common causes of death, contributing to more than four out of five CVD deaths. According to the World Health Organisation (WHO), one-third of these deaths occur prematurely in people under 70 years of age and are preventable [1]. To reduce the burden of CVD, secondary cardiovascular prevention mechanisms including healthy eating, physical activity, smoking cessation, reduction of alcohol consumption, maintaining healthy body weight and prescription and taking of appropriate pharmacotherapeutic treatment are essential. Secondary cardiovascular prevention programs are any strategies implemented to prevent or reduce the recurrence of further cardiovascular events [2] and these strategies have been proven to reduce the risk of cardiac event recurrence and improve clinical outcomes of patients [3]. Cardiac rehabilitation (CR) programs are one of the secondary cardiovascular prevention mechanisms.

CR is a structured secondary CVD prevention strategy defined by WHO as “the coordinated sum of activities required to influence favourably the underlying cause of cardiovascular disease, as well as to provide the best possible physical, mental, and social conditions, so that the patients may, by their own efforts, preserve or resume optimal functioning in their community and through improved health behaviour, slow or reverse progression of disease” [4]. One of the core components of a CR program is medication education which allows patients to understand their conditions, prescribed medication and promote adherence for improved outcomes. Adherence to therapy is defined by WHO as “the extent to which a person's behavior – taking medication, following a diet, and/or executing lifestyle changes, corresponds with agreed recommendations from a health care provider” [5]. Taking the prescribed medication at the right dose, frequency, duration and time is challenging especially for patients prescribed with multiple medications for multiple comorbidities.

Patients with CVD are mostly prescribed five or more long-term medications, also referred to as polypharmacy [6]. Poor medication adherence reduces quality of life of patients, increases disease prevalence, complications and health costs [7]. Implementing strategies that improve medication adherence such as CR is more impactful on the population's health than any medical technology advances and specific medical treatments [5]. However, the small studies conducted to assess the effectiveness of CR on medication adherence are not well established [8]. Considering medication adherence is one of the cornerstones for better health outcomes and CR programs are implemented to achieve this, it is critical to summarize the existing evidence, to affirm or not, the strategy for implementation of clinically effective strategies into practice. Therefore, we systematically summarised the available evidence on the effectiveness of CR programs versus standard care on medication adherence in patients with CVD and provide and recommend clinically transferable evidence for clinical practice.

A preliminary search of PROSPERO, MEDLINE, the Cochrane Database of Systematic Reviews, and *JBI Evidence* Synthesis was conducted, and no current or in-progress systematic reviews on the topic were identified.

## 2 Methods

This systematic review and meta-analysis was conducted according to JBI methodology for systematic reviews of effectiveness [9] and registered in PROSPERO [CRD42021284705]. Further details on, search, selection, extraction, appraisal, risk of bias assessments and effect measures were described in the published protocol [10].

### 2.1 Inclusion criteria

This study included adults ≥18 years of age with CVD and eligible to attend CR programs. Randomised control trials and quasi-experimental studies that measured the effectiveness of CR programs, regardless of types of core components, duration, or frequency, on medication adherence compared to medication adherence in standard care were included.

The primary outcome of this review was medication adherence. Studies that measured medication adherence as their primary or secondary outcomes using validated tools were considered. Studies that categorised adherence as high/moderate/low adherence were analysed in our study by considering moderate and low as low adherence. Data from studies that assessed mortality, hospital admission, primary care and emergency department visits, blood pressure, lipid profiles, and quality of life were also extracted as the secondary outcomes of this review if reported.

### 2.2 Search strategy and selection

Published and unpublished randomised controlled trials and quasi-experimental studies were searched on MEDLINE (via Ovid), Emcare (via Ovid), Embase (via Ovid), Cochrane CENTRAL, Scopus (via Elsevier), CINAHL (via EBSCO); and ClinicalTrials.gov and ProQuest Dissertations and Theses Global, TROVE, Networked Digital Library of Theses and Dissertations (NDLTD), World Health Organization International Clinical Trial Registry Platform, National Institute for Health and Care Excellence and Google scholar with no language restriction. Due to translation resource limitations, only studies published in English and Spanish were included. Studies published from database inception to the 9^th^ of Feb 2022 were included. Following search date, we have set an alert system on the databases searched, and up to this date, no major study on the topic has been published since then.

Following database searches, all citations were collated and uploaded into EndNote version 20.0 (Clarivate Analytics, PA, USA) and duplicates were removed. The full citations were then imported into the JBI System for the Unified Management, Assessment, and Review of Information (JBI SUMARI; JBI, Adelaide, Australia) [11] for the title and abstract and full-text screening. At all stages, screening was performed by two independent reviewers (LG, SC, KN, VP, JR, HD, NB, SS, OS, and MAP) and disagreement was resolved by involving a third reviewer (RC). Results of the search, study selection and inclusion process were reported and presented in a Preferred Reporting Items for Systematic Reviews and Meta-analyses (PRISMA-2020) flow diagram [12].

### 2.3 Assessment of methodological quality, data extraction and synthesis

Critical appraisal of eligible studies was performed by two independent reviewers (LG, SC, KN, VP, JR, HD, NB, SS, OS, and MAP) using standardized critical appraisal instruments from JBI for randomised control trials and quasi-experimental studies [9]. Any disagreement was resolved through discussion. When required, authors of papers were contacted for missing or additional information.

Two independent reviewers (LG, SC, KN, VP, JR, HD, NB, SS, OS, and MAP) extracted data from all the included studies using the standardized JBI data extraction tool [11]. Disagreements that arose were resolved through discussion.

A statistical meta-analysis of included studies was pooled using RevMan version 5.4.1 (Copenhagen: The Nordic Cochrane Centre, Cochrane) when possible. In our meta-analysis, the random effect model was used throughout. The effect size was presented in risk ratio (RR) (for dichotomous) or weighted/standardised mean difference (for continuous) and a 95 % confidence interval was calculated. Subgroup sensitivity analysis for medication adherence tools, modes of CR delivery (face to face, remote, face to face combined with remote delivery), health professional who delivered CR (pharmacist-led versus other health professionals), settings of service delivery (health facility based, home/community based, remote delivery) and follow up durations (≤3 months, 3 to 6-months and ≥12-months) was conducted. Patient characteristics were analysed using IBM SPSS statistics version 27 (Chicago, USA). The heterogeneity of studies was presented as standard χ^2^ and *I*^2^ tests. Publication bias was assessed using a funnel plot. Further investigation on publication bias was performed using random-effects model regression-based Harbord test for small study effect. When pooling of statistical meta-analysis was not possible after contacting the authors for missing data, results were presented in a narrative form in tables or figures. The longest duration of follow-up was used for the analysis in studies with more than one follow-up time.

### 2.4 Assessing certainty in the findings

Certainty of findings was presented using the Grading of Recommendations, Assessment, Development and Evaluation (GRADE) approach [13] and a Summary of Findings was created using GRADEpro GDT (McMaster University, ON, Canada).

## 3 Results

### 3.1 Study and participant characteristics

**Fig. 1:**
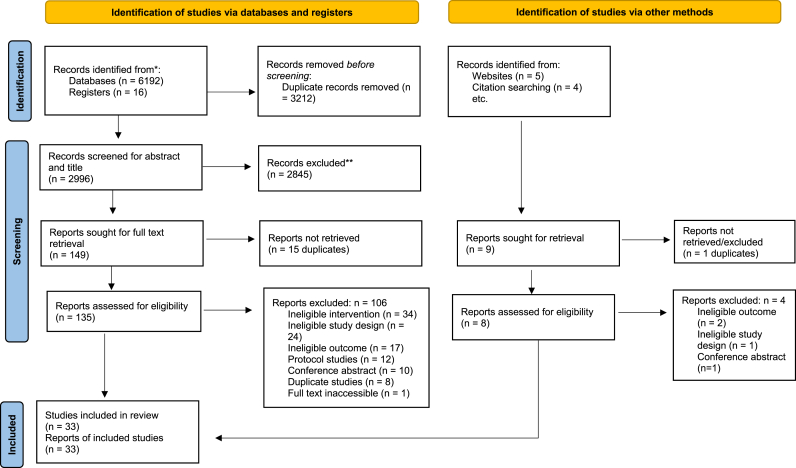
PRISMA 2020 flow diagram of process of search, selection, and inclusion of studies [12].

From 143 studies, 33 studies were included and analysed as detailed in the PRISMA chart (Fig. 1). The total number of participants were 16,677 ranging from 33 to 3595 per study with a mean (standard deviation, SD) of 505.4 (824.6) participants. The mean (SD) number of participants in the standard care and CR group were 235.1 (376.2) (range 16–1989), and 243.7 (396.0) (range 17–1970), respectively. The mean (SD) age of the standard care group did not significantly differ from the CR program group (64.5 (8.5) vs 64.1 (8.6) years; p = 0.851). Male participants were the dominant participants in both groups constituting 72.8 % and 73.4 % in the standard care, and the CR group, respectively. Most of the studies (27/33, 82 %) were published in the past ten years (2012–2022), and 7 of the 33 studies were conducted in the United States of America followed by China with 5 studies. All the included studies were randomized controlled trials, except one quasi-experimental. The duration of the interventions ranged from 7 to 1095 days with a mean (SD) duration of 220.8 (205.9) days. Heart failure (6 studies) and myocardial infarction (5 studies) were the common diagnoses in the included studies (Supplementary Table 1). As per the JBI appraisal tool for randomised control trials and quasi-experimental studies, the quality of the included studies was medium to high ranging 61.5 %–100 % (Supplementary Table 2).

### 3.2 Outcomes

#### 3.2.1 Medication adherence

**Fig. 2:**
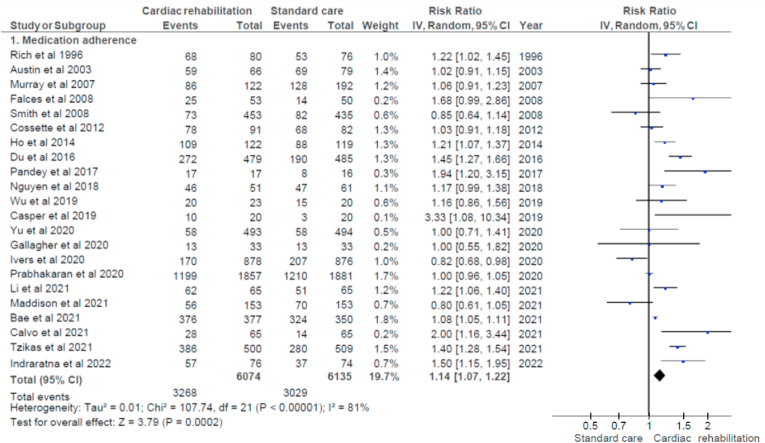
Forest plot of studies reported high medication adherence in the cardiac rehabilitation program and the standard care [[14], [15], [16], [17], [18], [19], [20], [21], [22], [23], [24], [25], [26], [27], [28], [29], [30], [31], [32], [33], [34], [35]].

**Fig. 3:**
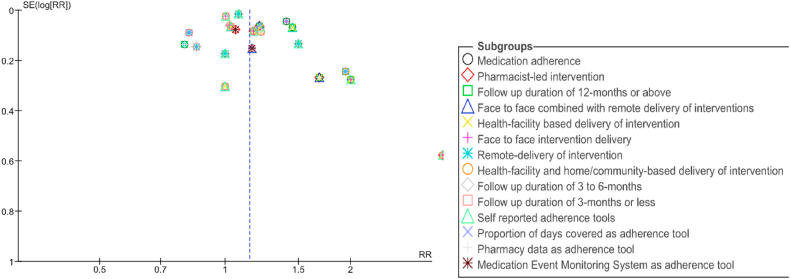
Funnel plot for medication adherence with subgroup sensitivity analysis factors.

Medication adherence was measured using different tools in the included studies. Of the 33 studies, 16 used validated self-reported tools to assess adherence (Supplementary Table 1). Of the 22 studies [[14], [15], [16], [17], [18], [19], [20], [21], [22], [23], [24], [25], [26], [27], [28], [29], [30], [31], [32], [33], [34], [35]] that reported medication adherence as high/good and low/poor or high/moderate/low adherence, using the random effect model, an overall increase in effect size of 14 % was observed in the CR group compared to standard care (RR = 1.14; 95 % CI: 1.07 to 1.22; p = 0.0002). These studies had very high heterogeneity (I^2^ estimate of 81 %, Fig. 2) which makes the pooled estimate less reliable. Investigation for publication bias using a funnel plot (Fig. 3), showed significant small study effect (p = 0.03). However, when the outlier study [17] with the same result in both groups was removed, no significant small study effect was observed (p = 0.16). The certainty of evidence for medication adherence was graded as low (Supplementary Table 3).

Sensitivity analysis for medication adherence was investigated. CR follow up durations of 12-months and longer showed an overall effect size increase of medication adherence by 34 % (RR = 1.34; 95 % CI: 1.14 to 1.57; p = 0.0003; I^2^ = 75 %) followed by face to face combined with remote delivery by 22 % (RR = 1.22; 95 % CI: 1.09 to 1.36; p = 0.0006; I^2^ = 59 %), pharmacist-led CR programs by 19 % (RR = 1.19; 95 % CI: 1.04 to 1.35; p = 0.009; I^2^ = 43 %) and health facility-based delivery of CR programs by 18 % (RR = 1.18; 95 % CI: 1.08 to 1.29; p = 0.0003; I^2^ = 80 %) (Supplementary Fig 1).

The remaining 11 studies [[36], [37], [38], [39], [40], [41], [42], [43], [44], [45]] which reported medication adherence as mean and SD were not included in the meta-analysis due to variations in the data reported, adherence tools used and how results were interpreted. Ten studies [[36], [37], [38], [39], [40], [41], [42], [43], [44]] reported mean medication adherence with an overall improvement observed in the CR group compared to standard care (Supplementary Table 4). While one study presented only % of adherence by medication class [45].

#### 3.2.2 Mortality

**Fig. 4:**
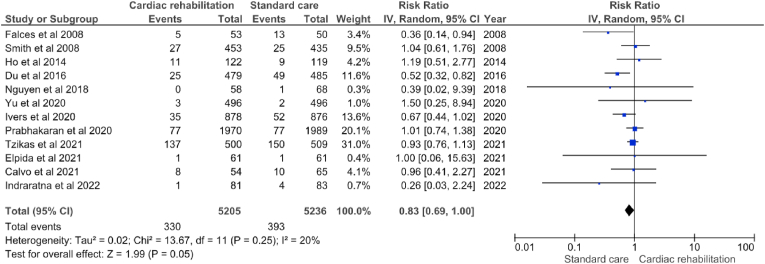
Forest plot of mortality in the cardiac rehabilitation program and standard care group [16,19,20,[22], [23], [24],^28^,^30^,^32^,^33^,^35^,^38^].

Twelve studies reported mortality data [16,19,20,[22], [23], [24],^28^,^30^,^32^,^33^,^35^,^38^]. Although statistically not significant-borderline, CR program participants had 17 % less relative risk of dying compared to standard care (RR = 0.83; 95 % CI: 0.69 to 1.00; p = 0.05) with an I^2^ estimate of 20 % (Fig. 4). The certainty of evidence for mortality was graded as high (Supplementary Table 3).

#### 3.2.3 Primary care and emergency department visits

Four studies reported emergency department or primary care visits [14,16,19,27] where two of these studies [14,16] missed required data and were not included in the forest plot (Supplementary Table 1). Participants enrolled in the CR program were less likely to visit their primary care or emergency department (Standardised Mean difference (SMD) = -0.19; 95 % CI: −0.30 to −0.08; p = 0.0008) than those who attended standard care with an I^2^ estimate of 0 % (Supplementary Fig 2). The certainty of evidence for primary care and emergency department visit was graded as high (Supplementary Table 3).

#### 3.2.4 Hospital admissions

Nine studies [14,16,20,23,27,28,30,35,38] reported data on number or rate of hospital admissions. Pooled estimates could not be presented due to the variability in the data reported, missing data and associated differences in interpretations. As summarised in Supplementary Table-5, an overall reduction in hospital admissions were observed in the CR program participants compared with the standard care gvel (Supplementary Table-3). Wroup.

#### 3.2.5 Quality of life

Nine studies [14,20,21,25,30,37,38,43,44] reported on quality-of-life data and six studies [14,20,25,38,43,44] were pooled in the statistical analysis. Participants enrolled in the CR program had a better quality of life than those who attended standard care, excluding an outlier study [38], (SMD = 0.93, 95 % CI: 0.38 to 1.49; p = 0.0010) with an I^2^ estimate of 91 %, very high heterogeneity indicating less reliability on the pooled estimate (Supplementary Fig 3A) with certainty of evidence graded as low level (Supplementary Table-3). When the outlier study [38] was included in the forest plot, the change in quality of life was non-significant (SMD = 0.57; 95 % CI: −0.23 to 1.37; p = 0.16) and the I^2^ estimate increased to 96 % (Supplementary Fig 3B). The other three studies [21,30,37] were not included in the plot as the data reported was not suitable for analysis (Supplementary Table 1). One study [30] reported the visual analogue scale results for quality of life where lower was interpreted as better quality of life and the other two studies [21,37] reported quality of life as a summary of the physical and mental component not the overall change in the quality of life. Better quality of life scores was observed in the CR group in both components compared to standard care.

#### 3.2.6 Lipid profile levels

Seven studies [15,17,19,22,36,37,43] presented data on low-density lipoprotein cholesterol (LDL-C) but two studies [15,17] were excluded from the meta-analysis due to the absence of required data (Supplementary Table 1). Three studies reported on the levels of high-density lipoprotein cholesterol (HDL-C) [19,36,37], and total cholesterol (TC) [17,36,37]. There was no significant difference in the level of LDL-C (SMD = −0.05; 95 % CI: −0.35 to 0.25; p = 0.74; I^2^ = 88 %) (Supplementary Fig 4A) and HDL-C (SMD = 0.44; 95 % CI: −0.12 to 0.99; p = 0.12; I^2^ = 94 %) (Supplementary Fig 4B), except for TC where a mean reduction of 0.26 (SMD = −0.26; 95 % CI: −0.44 to −0.07; p = 0.006; I^2^ = 0 %) was observed in the CR compared with the standard care group (Supplementary Fig 4C). The certainty of this evidence was graded as high (Supplementary Table-3). Only one study [37] reported on triglyceride levels and the CR program resulted in an approximate mean reduction of 16 mg/dL (149.33 vs 165.27) compared with the standard care.

#### 3.2.7 Blood pressure levels

Eight studies [15,17,19,21,22,35,36,43] reported on systolic blood pressure (SBP), but one study [15] with missing data was excluded. Five studies [17,21,22,35,36] presented data on diastolic blood pressure (DBP) with one study lacking data and excluded from the analysis [17]. No significant difference was observed in both systolic (SMD = −0.02; 95 % CI: −0.19 to 0.14; p = 0.77; I^2^ = 66 %) (Supplementary Fig 5A) and diastolic blood pressure (SMD = 0.01; 95 % CI: −0.09 to 0.12; p = 0.79; I^2^ = 0 %) (Supplementary Fig 5B**)** in the CR programs compared with the standard care group.

## 4 Discussion

Our systematic review assessed the effectiveness of CR programs versus standard care on medication adherence in patients with cardiovascular disease. Although CR programs are based on the best available evidence and recommended by clinical practice guidelines, the effectiveness of these programs on medication adherence, one of the core components of CR, has not been well established. Our systematic review and meta-analysis is the first one to show that CR programs are effective in:

• improving medication adherence by an overall effect size of 14 % with high study heterogeneity
• reducing the risk of mortality by 17 %
• reducing visits to primary care and emergency departments by a mean difference of 0.19
• improving quality of life of patients by a mean difference of 0.93
• reducing total cholesterol by a mean difference of 0.26.
• but no statistically significant difference was observed in LDL-C, HDL-C, SBP and DBP levels

These findings were supported by another study where multi-disciplinary teams delivered bundled interventions [46], such as CR programs as in our case, improved medication adherence. A systematic review and meta-analysis by Torres-Robles et al. reported education plus technical support were more effective (OR = 0.44; 95 % credibility interval: 0.26 to 0.73) compared with standard care in improving medication adherence in patients with cardiovascular and metabolic diseases. They were also ranked as the best intervention (79.6 %) as per the probability ranking analysis [47].

High heterogeneity (I^2^ of 81 %) was observed in the studies that measured medication adherence within CR programs. Some potential reasons include the difference in tools used to measure medication adherence. Most of the tools used in the included studies were self-reported assessment tools. These tools differed in the numbers and content of questions to assess medication adherence. Other tools were used to provide data on pharmacy refill data, pill count method, electronic monitoring device mainly medication event monitoring system, medication possession ratio, the proportion of days covered, and serum concentration assay of medications. We have conducted a sensitivity analysis of the different tools used by the included studies in our review. Heterogeneity remains high with the studies (n = 11) that used self-reported tools (I^2^ = 78 %), pharmacy data (I^2^ = 78 %), and proportion of days covered (I^2^ = 89 %), but low for studies (n = 2) that used medication event monitoring systems (I^2^ = 0 %) (Supplementary Fig 1). This high heterogeneity might be related to the difference in the numbers and components of the questions in the tools, variability of the pharmacy data, and proportion of days covered and how they were measured. Heterogeneity could be further explained by the variability of the interpretation of the scores of the same tool, reporting and interpretation of the data [17,18,21,25]. The cut-points for high adherence were also variable with the majority of the studies stating greater than or equal to 80 % as high adherence while others used greater than or equal to 95 % [20] or 85 % [14] and reporting of direct interpretation of total scores of the tools used [23,25,30,31,35]. The influencing factors mentioned here are supported by a large-sized systematic review and meta-analysis that detailed the effect size of the risk of bias; in particular, the various adherence tools used where the effect size for adherence measures was described for pharmacy refill as 0.261, pill count as 0.361, and self-reported as 0.261 [48]. Although the same study described the effect size of the risk of bias for medication event monitoring system measure was 0.349 [48], our review and meta-analysis showed no heterogeneity.

Other factors that could contribute to the variability in medication adherence and heterogeneity were the difference in modes of delivery (face to face, remote, and combination of face to face with remote), settings (facility-based, home/community-based, or remote), duration of the program (≤3-months, 3 to 6-months and ≥12-months), and the personnel who provided the intervention (Supplementary Table-1 and supplementary Fig 1). Our systematic review has shown that CR programs with longer follow up durations (≥12-months and longer) delivered face to face combined with remote modes of delivery provided in health facility settings led by a pharmacist showed an overall effect size increase of medication adherence ranging from 18 % to 34 %. Our findings were supported by a study which showed that face to face mode of interventions (effect size of 0.331) delivered by a pharmacist (effect size 0.337) that targeted individual patients/family focus (effect size of 0.302) provided in health care clinic (effect size of 0.323) were more effective than delivering through other modes of delivery, or by other health professionals which targeted health care providers/health systems or in a home setting, respectively [48]. Our meta-analysis showed that heterogeneity was high for face to face (I^2^ = 87 %), remote (I^2^ = 78 %), and face to face combined with remote delivery (I^2^ = 59 %). Similarly, heterogeneity was high for health facility-based delivery (I^2^ = 80 %) and health facility combined with home/community-based delivery (I^2^ = 65 %). Sensitivity analysis for follow up duration showed similarly high heterogeneity where follow up duration of 12-months or longer showed an I^2^ estimate of 75 %, 3 to 6-months an I^2^ estimate of 60 %, and I^2^ estimate of 56 % for follow up duration of 3-months or shorter (Supplementary Fig 1). The high heterogeneity resulted in the low degree of certainty of evidence in the GRADE report summary of finding for medication adherence and quality of life as the factors contributing to variation were similar (Supplementary Table 3). Considering the high level of heterogeneity among the included studies for medication adherence and quality of life, the results should be interpreted with caution as the certainty of evidence level was graded low. However, the effect of CR on reduction of mortality, primary and emergency department visits and total cholesterol level were significant with high degree of evidence certainty.

## 5 Strengths and limitations of the study

The strength of our study was searching for published and unpublished literature on the topic of interest specifically randomised controlled trials and quasi-experimental studies. The main limitation of our study was the high heterogeneity of the data reported by the studies as they have used different tools of medication adherence measures and cut-off points of high/good adherence, the scoring and interpretation of the data. Also, the variability of CR program interventions, contents, follow up duration and personnel delivering CR programs have contributed to the heterogeneity. Although our search was not limited by language, only papers published in English and Spanish were screened and analysed due to financial constraints for translation services.

## 6 Conclusion and recommendations

Cardiac rehabilitation programs significantly improve medication adherence compared with standard care in patients with CVD. CR programs also reduce the risk of mortality, hospital admissions, visits to primary care and emergency departments and improve patients’ quality of life. Usage and availability of the same validated medication adherence tool throughout CR programs is strongly recommended to effectively measure effectiveness of the program. Assessing the prescriptions of evidence-based pharmacotherapy in patients with cardiovascular disease within CR programs might provide more insight into why changes in LDL-C, HDL-C and blood pressure levels were not significant with improved medication adherence.

## Author contribution

LG, SC, KN, VP, JR, HD, NB, SS, OS, and MAP were involved in screening, data extraction and critical appraisal of the review. LG drafted the manuscript. AG provided statistical advice. All authors provided advice and assistance in formulating the review protocol, critically reviewing and editing the manuscript.

## Systematic review protocol

PROSPERO CRD42021284705 registered and published in JBI Evid Synth 2022; 20 (12):2986–2994.

## Funding

RC and JH were supported in the development of this manuscript by the 10.13039/501100001785Flinders University Caring Futures Institute (Cardiac 10.13039/501100014065Focus Area Research Grant 2021), Australia. An 10.13039/100004325AstraZeneca fellowship, Australia, supports LG. AstraZeneca did not have input in any aspect of this work.

## Conflicting of interests

Authors declare no conflict of interest.
