# Supplementary material for: Effectiveness of cardiac rehabilitation programs on medication adherence in patients with cardiovascular disease: A systematic review and meta-analysis
Source: Int J Cardiol Cardiovasc Risk Prev. 2023 Dec 12;20:200229. doi: 10.1016/j.ijcrp.2023.200229 (PMC10770721; doi:10.1016/j.ijcrp.2023.200229)
Supplement: Multimedia component 1 [file mmc1.docx]

| **Study**  Supplementary Table-1: Study and patient characteristics of included papers (N=33) | **Diagnosis** | **Total population (N)** | **Total population (N)** | | **Age (years)** | | **Male (mean value)** | | **Intervention type/s** | **Settings** | **Medication adherence tool used** | **Follow up duration** | **Country** |
| --- | --- | --- | --- | --- | --- | --- | --- | --- | --- | --- | --- | --- | --- |
|  |  |  | **SC** | **CR** | **SC** | **CR** | **SC** | **CR** |  |  |  |  |  |
| Austin *et al.* 2003 [14] | Heart failure | 200 | 100 | 100 | 71.8 | 71.9 | 66 | 66 | education and patient and family support on clinical management | Hospital/Community/outpatient clinic-based | Serum assay | 24-wk | UK |
| Bae *et al.* 2021 [15] | Coronary heart disease undergoing percutaneous coronary intervention | 879 | 439 | 440 | 60.7 | 60.1 | 364 | 368 | Access to website and SMS text messages regarding lifestyle modifications | University teaching hospital | Modified Morisky Scale (MMS) | 6-mo | Korea |
| Bennett *et al.* 2019 [45] | Myocardial Infarction, Percutaneous Coronary intervention, Angina, Hypertension, Hyperlipidemia | 41 | 17 | 24 | 56.1 | 55.4 | 13 | 16 | telephone-based brief motivational interviewing (MI) counselling session | urban safety net hospital Medical Center | Pharmacy fill data | 5-wk | USA |
| Calvo *et al.* 2021 [16] | myocardial infarction undergoing percutaneous coronary intervention | 143 | 75 | 68 | 81.6 | 82.9 | 54 | 30 | Nursing health education intervention | a tertiary care hospital | 4 item-Morisky-Green Levine Medication Adherence Scale and Haynes-Sackett method | 12-mo | Spain |
| Casper *et al.* 2019 [17] | Acute Coronary Syndrome | 40 | 20 | 20 | 54.5 | 52.3 | 15 | 16 | standard medical care plus clinical pharmacist–provided services. | University hospitals | Arabic version of the 8‐item Morisky Adherence Questionnaire | 3-mo | Egypt |
| Dale *et al.* 2015 [36] | Coronary heart disease (myocardial infarction, angina or revascularisation) | 123 | 62 | 61 | 59.9 | 59.0 | 52 | 48 | text message and a supporting website | at home/community settings | Morisky 8-item Medication Adherence Questionnaire. | 6-mo | New Zealand |
| Falces *et al.* 2008 [20] | heart failure | 103 | 50 | 53 | 80.1 | 79.0 | 22 | 21 | Pharmacist-led personal interview at the time of discharge and subsequent telephone reinforcement. | Hospital | Pill counting method | 6, and 12-mo | Spain |
| Pakpour *et al.* 2017 [37] | Patients who underwent coronary artery bypass graft (CABG) surgery | 288 | 144 | 144 | 75.2 | 74.3 | 94 | 97 | Psychoeducation, motivational interviewing, and short message services on medication adherence, quality of life (QoL), and mortality rates | at home/community settings | The Medication Adherence Rating Scale (MARS) Likert scale and the pharmacy refill rate | 6, 12, and 18-mo | Iran |
| Cossette *et al.* 2012 [18] | Acute Coronary Syndrome | 242 | 121 | 121 | 59.4 | 59.4 | 109 | 98 | a face-to-face meeting before discharge; a telephone call 3 days post-discharge and a telephone call or a hospital meeting 10 days post- | a specialized cardiac hospital | The 4-item Self-Reported Medication Taking Scale. | 6-wk | Canada |
| Du *et al.* 2016 [19] | Acute Coronary Syndrome who underwent percutaneous coronary intervention | 964 | 485 | 479 | 61.6 | 60.4 | 531 | 349 | Telephone calls and medical consultations from cardiologists | Hospital based | Morisky–Green questionnaire. | 12 and 36-mo | China |
| Elpida *et al.* 2021 [38] | Heart failure | 122 | 61 | 61 | 69.2 | 65.9 | 52 | 50 | (a) orientation, (b) elicitation, (c) restructuring, (d) application, and (e) review of education sessions on the core components | Hospital based | The Self Efficacy for Appropriate Medication use Scale | 3 and 6-mo | Greece |
| Gallagher *et al.* 2020 [21] | Atrial fibrillation | 72 | 36 | 36 | 66 | 63 | 20 | 20 | Nurse-led face-to-face education and risk factor management session with 4 follow-up telephone calls. | Both inpatient and outpatient clinics | Morisky Medication Adherence Scale (MMAS) | 3-mo | Australia |
| Goodyer *et al.* 1995 [39] | Heart failure | 100 | 50 | 50 | 85 | 84 | 12 | 15 | Pharmacist-led intensive medication counselling | outpatient clinics | Tablet count | 3-mo | UK |
| Maddison *et al.* 2021 [26] | Acute Coronary Syndrome (including those who had undergone a percutaneous coronary revascularization procedure) | 306 | 153 | 153 | 61 | 61 | 113 | 123 | a personalized, automated program of self-management that was delivered via SMS text messages over 24 weeks | Hospital based | medication possession ratio (MPR) (***used in our review analysis)*** and Morisky 8-item Medication Adherence Scale. | 24 and 52-wk | New Zealand |
| Nguyen *et al.* 2018 [28] | Acute Coronary Syndrome | 166 | 87 | 79 | 59.8 | 62 | 59 | 61 | Pharmacist-led educational and behavioural interventions | Inpatient and outpatient clinics | The 8–item Morisky Medication Adherence Scale | 1 and 3-mo | Vietnam |
| Park *et al.* 2013 [40] | Coronary artery disease | 58 | 30 | 28 | 58.3 | 57.9 | 25 | 23 | Individual face-to-face education using a tailored resource package and telephone-delivered health coaching | a tertiary care cardiac centre | self-report questionnaires | 12-wk | Korea |
| Rich *et al.* 1996 [31] | Congestive heart failure | 156 | 76 | 80 | 78.4 | 80.5 | 31 | 21 | comprehensive patient education, dietary and social service consultations, medication review, and intensive post discharge follow-up | A University teaching hospital | Pill count. | 30-d | USA |
| Stamm-Balderjah *et al.* 2016 [41] | Coronary heart disease | 402 | 270 | 132 | 61.2 | 61.5 | 140 | 70 | Goal setting (IGb) at the end of the rehabilitation measure and Goal checking (IGa) three months after the end of the rehabilitation measure. ***IGa are analysed in this review*** | In the community | The Morisky adherence tool of 4-item | 6 and 12-mo | Germany |
| Tzikas *et al.* 2021 [33] | non-valvular Atrial Fibrillation | 1009 | 509 | 500 | 76 | 75.5 | 265 | 275 | Motivational interviewing, and tailored counselling on medication adherence | University hospital | The proportion of days covered (PDC). | 1-yr | Greece |
| Volpp *et al.* 2017 [42] | Acute myocardial infarction | 1503 | 503 | 1000 | 60.6 | 61.2 | 313 | 657 | an intervention using electronic pill bottles combined with lottery incentives and social support for medication adherence | Hospital and home-based | The proportion of days covered (PDC). | 1-yr | USA |
| Johnston *et al.* 2016 [43] | Myocardial infarction | 166 | 80 | 86 | 58.4 | 56.8 | 63 | 71 | a complete interactive patient support tool installed on patients own smartphones containing an extended drug adherence e-diary and secondary prevention modules | Home-based | The 5 items Medication Adherence Rating Scale (MARS- | 6-mo | Sweden |
| Murray *et al.* 2007 [27] | Heart failure | 314 | 192 | 122 | 62.6 | 61.4 | 65 | 39 | A pharmacist-led intervention using a protocol where medications were dispensed only with verbal/written instructions and monitored through a study database | Home based (telephone) | Medication Event Monitoring System (MEMS) | 3-mo | USA |
| Pandey *et al.* 2017 [29] | Myocardial infarction | 33 | 16 | 17 | 62.1 | 64.6 | 14 | 6 | Standard care plus a reminder text message of preferred time to take their medications. | Cardiac rehab centre and home based | The percentage of days covered (PDC). | 12-mo | Canada |
| Wu *et al.* 2019 [34] | Heart failure | 43 | 20 | 23 | 67 | 65 | 14 | 11 | access to a guide, a 60min in-person intervention session with a trained interventionist, and bi-weekly phone sessions to support their understanding of medications, and their conditions and management | Medical center | The Medication Event Monitoring System (MEMS) | 3-mo | USA |
| Yu *et al.* 2020 [35] | Patients who underwent isolated coronary artery bypass grafting (CABG) | 1000 | 499 | 501 | 57.1 | 57.4 | 422 | 433 | Received an advanced smartphone application for 6 months, designed to provide medication reminders and cardiac health education and encouragement and feedback. Also, weekly questionnaire about medication adherence, blood pressure and body mass index | Teaching hospital | Chinese version of the 8-item Morisky Medication Adherence Scale (MMAS-8) | 3 and 6-mo | China |
| Ho *et al.* 2014 [22] | Acute Coronary Syndrome | 241 | 119 | 122 | 64 | 63.8 | 116 | 120 | An appointment for an in-person visits or telephone consultation with a pharmacist | Medical centre | The proportion of days covered (PDC). | 1-yr | USA |
| Indraratna *et al.* 2022 [23] | Acute Coronary Syndrome or Heart failure | 164 | 83 | 81 | 61.7 | 61.3 | 65 | 65 | Received an app providing 3 weekly educational push notifications to promote healthy behaviour choices. | Hospital | Morisky-Green-Levine 4-item medication compliance (MGL) score | 6-mo | Australia |
| Ivers *et al.* 2020 [24] | Myocardial infarction | 2632 | 876 | 878 | 66.8 | 65.9 | 624 | 626 | Varying, tailored contents were delivered by mail and by telephone through 1) Mail-out reminders; 2) Mail out reminders+ telephone calls. ***Mail and phone call were analysed in this review***. | Cardiac centres | The mean proportion of days covered | 7-d | Canada |
| Li *et al.* 2021 [25] | patients who underwent heart valve replacement surgery | 130 | 65 | 65 | 53.6 | 53.8 | 38 | 39 | Nurse-led health education was given to patients every Friday, about 45 min each time and followed through WeChat, daily medication reminders | Hospital | Modified Morisky Scale with 4 items (MMS-4) | 2 and 6-mo | China |
| Prabhakaran *et al.* 2020 [30] | Acute myocardial infarction | 3959 | 1989 | 1970 | 53.4 | 53.4 | 1709 | 1699 | The Yoga-CaRe program involved 13 direct contact sessions spread over 12 weeks, involved a set of gentle yoga exercises, followed by a discussion on lifestyle and psychosocial concerns | Hospital and home based | Morisky Medication Adherence Scale-8 (MMAS-8) | 12-wk | India |
| Smith *et al.* 2008 [32] | Myocardial infarction | 888 | 435 | 453 | 65.04 | 64.7 | 287 | 311 | Two mailed communications of personalised letter followed by another similar letter and a brochure. Both mailings also included a wallet card with suggested questions to ask their clinician, space to list their medications, and space to record additional queries | Home and clinic based | proportion-of-days-covered (PDC). | 3 and 6-mo | USA |
| Xu *et al.* 2021 [44] | Acute myocardial infarction | 100 | 50 | 50 | 56.3 | 56.3 | 42 | 40 | The research group received the WeChat platform health management combined with refined continuous nursing model | University hospital | Medication possession ratio. | 3-mo | China |
| Zhao *et al.* 2015 [49] | Coronary heart disease | 90 | 45 | 45 | Stratified age group presented |  | 17 | 19 | clinical care plus pharmacist support that included medication review, education, lifestyle management, discharge guidance, and telephone follow-up | Hospital | Not available | 6-mo | China |
| **Total** |  | 16677 | 7757 | 8042 |  |  | **5646** | **5903** |  |  |  |  |  |

SC-standard care; CR-Cardiac rehabilitation programs; mo-month; Y-year; wk-week; d-day; N-number

Supplementary Table-2: Critical Appraisal summary of included studies (N=33)

Table-1: Critical Appraisal summary of included studies (N=33)

#### Quasi-Experimental Study

| **Citation** | **Q1** | **Q2** | **Q3** | **Q4** | **Q5** | | **Q6** | **Q7** | **Q8** | **Q9** | **Y %** |
| --- | --- | --- | --- | --- | --- | --- | --- | --- | --- | --- | --- |
| Park *et al* 2013^40^ | Y | Y | Y | Y | Y | | Y | Y | Y | Y | 100% |
| Q1. Is it clear in the study what is the 'cause' and what is the 'effect' (i.e., there is no confusion about which variable comes first)? | | | | | | Q2. Were the participants included in any comparisons similar? | | | | | |
| Q3. Were the participants included in any comparisons receiving similar treatment/care, other than the exposure or intervention of interest? | | | | | | Q4. Was there a control group? | | | | | |
| Q5. Were there multiple measurements of the outcome both pre and post the intervention/exposure? | | | | | | Q6. Was follow up complete and if not, were differences between groups in terms of their follow up adequately described and analyzed? | | | | | |
| Q7. Were the outcomes of participants included in any comparisons measured in the same way? | | | | | | Q8. Were outcomes measured in a reliable way? | | | | | |
| Q9. Was appropriate statistical analysis used? | | | | | |  | | | | | |

#### Randomized Controlled Trial

| **Citation** | **Q1** | **Q2** | **Q3** | **Q4** | **Q5** | **Q6** | **Q7** | **Q8** | **Q9** | **Q10** | **Q11** | **Q12** | **Q13** | **Y %** |
| --- | --- | --- | --- | --- | --- | --- | --- | --- | --- | --- | --- | --- | --- | --- |
| Austin *et al* 2003 [14] | Y | Y | Y | Y | N | U | Y | Y | Y | Y | Y | Y | Y | 84.6 |
| Bae *et al* 2021 [15] | Y | Y | Y | N | Y | Y | Y | Y | Y | Y | Y | Y | Y | 92.3 |
| Clark *et al* 2017 [45] | Y | U | Y | U | Y | U | Y | Y | Y | Y | Y | Y | Y | 76.9 |
| Calvo *et al* 2021 [16] | Y | N | N | Y | Y | U | U | Y | Y | Y | Y | Y | Y | 69.2 |
| Casper *et al* 2019 [17] | Y | Y | Y | Y | Y | U | Y | Y | Y | Y | Y | Y | Y | 92.3 |
| Dale *et al* 2015 [36] | Y | Y | U | N | Y | N | Y | Y | Y | Y | Y | Y | Y | 76.9 |
| Falces *et al* 2008 [20] | U | U | Y | U | U | U | Y | Y | Y | Y | Y | Y | Y | 61.5 |
| Cossette *et al* 2012 [18] | Y | Y | Y | Y | Y | Y | Y | Y | Y | Y | Y | Y | Y | 100 |
| Ho *et al* 2014 [22] | Y | Y | Y | N | N | U | Y | Y | Y | Y | Y | Y | Y | 76.9 |
| Ivers *et al* 2020 [24] | Y | Y | Y | N | N | Y | U | Y | Y | Y | Y | Y | Y | 76.9 |
| Prabhakaran *et al* 2020 [30] | Y | Y | Y | N | N | N | Y | Y | Y | Y | Y | Y | Y | 76.9 |
| Murray *et al* 2007 [27] | Y | Y | Y | N | N | N | Y | Y | Y | Y | Y | Y | Y | 76.9 |
| Li *et al* 2021 [25] | Y | U | Y | N | N | U | Y | Y | Y | Y | Y | Y | Y | 69.2 |
| Indraratna *et al* 2022 [23] | Y | Y | Y | N | N | N | Y | Y | Y | Y | Y | Y | Y | 76.9 |
| Smith *et al* 2008 [32] | Y | Y | Y | N | N | Y | U | Y | Y | Y | Y | Y | Y | 76.9 |
| Pandey et al 2017 [29] | Y | U | Y | N | N | U | Y | Y | Y | Y | Y | Y | Y | 69.2 |
| Xu *et al* 2021 [44] | Y | N | Y | N | N | N | Y | Y | Y | Y | Y | Y | Y | 69.2 |
| Zhao *et al* 2015 [49] | Y | N | Y | N | N | N | Y | Y | Y | Y | Y | Y | Y | 69.2 |
| Johnston *et al* 2016 [43] | Y | U | Y | N | N | N | N | Y | Y | Y | Y | Y | Y | 61.5 |
| Gallagher *et al* 2020 [21] | Y | U | Y | Y | U | Y | Y | Y | Y | Y | Y | Y | Y | 84.6 |
| Goodyer *et al* 1995 [39] | Y | Y | Y | U | U | Y | Y | Y | Y | Y | Y | Y | Y | 84.6 |
| Pakpour *et al* 2017 [37] | Y | N | Y | N | U | Y | Y | Y | Y | Y | Y | Y | Y | 76.9 |
| Du *et al* 2016 [19] | Y | Y | Y | U | Y | Y | Y | Y | Y | Y | Y | Y | Y | 92.3 |
| Elpida *et al* 2020 [38] | Y | N | Y | U | U | U | Y | Y | Y | Y | Y | Y | Y | 69.2 |
| Nguyen *et al* 2018 [28] | Y | Y | Y | N | N | Y | Y | Y | Y | Y | Y | Y | Y | 84.6 |
| Rich *et al* 1996 [31] | Y | Y | Y | Y | Y | Y | Y | Y | Y | Y | Y | Y | Y | 100 |
| Stamm-Balderjah *et al* 2016 [41] | Y | N | Y | N | N | N | Y | Y | Y | Y | Y | Y | Y | 69.2 |
| Tzikas *et al* 2021 [33] | Y | N | Y | N | N | Y | Y | Y | Y | Y | Y | Y | Y | 76.9 |
| Maddison *et al* 2021 [26] | Y | N | Y | N | Y | Y | Y | Y | Y | Y | Y | Y | Y | 84.6 |
| Volpp *et al* 2017 [42] | Y | N | Y | Y | N | Y | Y | Y | Y | Y | Y | Y | Y | 84.6 |
| Wu *et al* 2019 [34] | Y | Y | Y | N | N | U | Y | Y | Y | Y | Y | Y | Y | 76.9 |
| Yu *et al* 2020 [35] | Y | N | Y | N | N | N | Y | Y | Y | Y | Y | Y | Y | 69.2 |

Y-yes; N-no, U-unclear

| Q1. Was true randomization used for assignment of participants to treatment groups? | Q2. Was allocation to treatment groups concealed? |
| --- | --- |
| Q3. Were treatment groups similar at the baseline? | Q4. Were participants blind to treatment assignment? |
| Q5. Were those delivering treatment blind to treatment assignment? | Q6. Were outcomes assessors blind to treatment assignment? |
| Q7. Were treatments groups treated identically other than the intervention of interest? | Q8. Was follow up complete and if not, were differences between groups in terms of their follow up adequately described and analyzed? |
| Q9. Were participants analysed in the groups to which they were randomized? | Q10. Were outcomes measured in the same way for treatment groups? |
| Q11. Were outcomes measured in a reliable way? | Q12. Was appropriate statistical analysis used? |
| Q13. Was the trial design appropriate, and any deviations from the standard RCT design (individual randomization, parallel groups) accounted for in the conduct and analysis of the trial? |  |

| **Supplementary Table 3: Summary of findings** | | | | | | |
| --- | --- | --- | --- | --- | --- | --- |
| **Effectiveness of cardiac rehabilitation program vs standard care on medication adherence in patients with cardiovascular disease** | | | | | | |
| **Patient or population:** patients with cardiovascular disease  **Intervention:** Cardiac rehabilitation programs  **Comparison:** standard care  **Outcomes:** Medication adherence; Mortality; Primary care and or emergency department visit; Quality of life; Low density lipoprotein cholesterol; High density lipoprotein cholesterol; Total cholesterol | | | | | | |
| Outcomes | **Anticipated absolute effects^*^** (95% CI) | | Relative effect (95% CI) | № of participants (studies) | Certainty of the evidence (GRADE) | Comments |
|  | **Risk with standard care** | **Risk with Cardiac rehabilitation programs** |  |  |  |  |
| **Medication adherence** assessed with: Self-assessment tools, serum concentration, Pill count method follow-up: range 7 days to 1095 days | 494 per 1,000 | **563 per 1,000** (548 to 583) | **RR 1.14** (1.11 to 1.18an o) | 12209 (22 RCTs) | ⨁⨁◯◯ Low^a,b^ | Certainty of evidence is low due to high heterogeneity and different measuring tools |
| **Mortality** follow-up: range 3 months to 21.6 months | 75 per 1,000 | **62 per 1,000** (52 to 75) | **RR 0.83** (0.69 to 1.00) | 10441 (12 RCTs) | ⨁⨁⨁⨁ High | High certainty of evidence |
| **Primary care and or emergency department visits** follow-up: range 9 months to 36 months | - | SMD **0.19 lower** (0.3 lower to 0.08 lower) | - | 1278 (2 RCTs) | ⨁⨁⨁⨁ High | High certainty of evidence |
| **Quality of life** assessed with self-assessment tools follow-up: range 3 months to 12 months | - | SMD **0.93 higher** (0.38 higher to 1.49 higher) | - | 674 (5 RCTs) | ⨁⨁◯◯ Low^b,c^ | Low certainty of evidence due to high heterogeneity and different measuring tools |
| **Low density lipoprotein cholesterol** (LDL-C) follow-up: range 6 months to 36 months | - | SMD **0.05 lower** (0.35 lower to 0.25 higher) | - | 1778 (5 RCTs) | ⨁⨁⨁◯ Moderate^d^ | Moderate certainty of evidence due to heterogeneity among studies |
| **High density lipoprotein cholesterol** (HDL-C) follow-up: range 6 months to 36 months | - | SMD **0.44 higher** (0.12 lower to 0.99 higher) | - | 1375 (3 RCTs) | ⨁⨁⨁◯ Moderate^d^ | Moderate certainty of evidence due to heterogeneity among studies |
| **Total cholesterol** follow-up: range 3 months to 18 months | - | SMD **0.26 lower** (0.44 lower to 0.07 lower) | - | 451 (3 RCTs) | ⨁⨁⨁⨁ High | High certainty of evidence |
| ***The risk in the intervention group** (and its 95% confidence interval) is based on the assumed risk in the comparison group and the **relative effect** of the intervention (and its 95% CI). **CI:** confidence interval; **OR:** odds ratio; **RR:** risk ratio; **SMD:** standardised mean difference | | | | | | |
| **GRADE Working Group grades of evidence** **High certainty:** we are very confident that the true effect lies close to that of the estimate of the effect. **Moderate certainty:** we are moderately confident in the effect estimate: the true effect is likely to be close to the estimate of the effect, but there is a possibility that it is substantially different. **Low certainty:** our confidence in the effect estimate is limited: the true effect may be substantially different from the estimate of the effect. **Very low certainty:** we have very little confidence in the effect estimate: the true effect is likely to be substantially different from the estimate of effect. | | | | | | |

#### Explanations

a. The outcome was assessed with various methods-self assessment tools, serum concentration and pill count method

b. The heterogeneity among the studies was high potentially contributing due to the variation of tools used to assess the outcome, follow-up duration, contents, and mode of delivery of the program

c. The outcome was measured with different self-assessment tools at the different duration of follow-up periods which might also be affected by the content and mode of delivery of the program (intervention) in participants with varying degrees of illness.

d. The Heterogeneity among the studies was high may be contributed to the difference in sample size, duration of follow-up, the severity of the patient's condition, content, and mode of delivery of the program (intervention).

Supplementary Table-4: Summary of studies that reported medication adherence as mean (N=10)

| **Study** | **Standard care number** | **High Adherence standard care** | **SD** | **CR number** | **High adherence CR** | **SD** |
| --- | --- | --- | --- | --- | --- | --- |
| Dale *et al* 2015 [36] | 62 | 6.8 | 1.2 | 61 | 7.3 | 0.9 |
| Pakpour *et al* 2017 [37] | 144 | 62.03 | 18.77 | 144 | 73.24 | 15.33 |
| Elpida *et al* 2021 [38] | 61 | 18.1 | 3.14 | 61 | 20.98 | 0.13 |
| Goodyer *et al* 1995 [39] | 50 | 51 | 31.5 | 50 | 93 | 11.7 |
| Park *et al* 2013 [40] | 30 | 53 | 7.52 | 28 | 56.14 | 5.11 |
| Stamm-Balderjah *et al* 2016 [41] | 201 | 0.17 | NA | 109 | 0.22 | NA |
| Volpp *et al* 2017 [42] | 337 | 0.42 | 0.39 | 682 | 0.46 | 0.39 |
| Johnston *et al* 2016* [43] | 77 | 22.8 | 41.3 | 85 | 16.6 | 43.9 |
| Xu *et al* 2021 [44] | 50 | 77.43 | 8.06 | 50 | 86.25 | 8.03 |
| Zhao *et al* 2015 [49] | 42 | 79.36 | 15.46 | 43 | 93.39 | 6.56 |

*Mean non-adherence reported; SD-standard deviation; NA-not available; CR-cardiac rehabilitation

Supplementary Table-5: Summary of hospital admission data reported by studies (N=9)

| **Study** | **SC number** | **Number/rate of hospital admission SC** | **SD** | **CR number** | **Number/rate of hospital admission CR** | **SD** | **HR** |
| --- | --- | --- | --- | --- | --- | --- | --- |
| Austin *et al* 2003 [14] | 94 | 0.35 | NR | 85 | 0.13 | NR | NR |
| Murray *et al* 2007 [27] | 192 | 0.97 | 1.78 | 122 | 0.78 | 1.66 | NR |
| Falces *et al* 2008 [20] | 50 | 1.12 | 1.96 | 53 | 0.55 | 0.91 | NR |
| Nguyen *et al* 2018 [28] | 68 | 6 | NR | 58 | 7 | NR | NR |
| Prabhakaran *et al* 2020 [30] | 1881 | 59 | NR | 1857 | 48 | NR | 0.82 (0.56 to1.20) |
| Yu *et al* 2020 [35] | 496 | 37 | NR | 496 | 26 | NR | NR |
| Calvo *et al* 2021 [16] | 65 | 0.56 | NR | 54 | 0.59 | NR | NR |
| Elpida *et al* 2021 [38] | 61 | 22 | NR | 61 | 8 | NR | NR |
| Indraratna *et al* 2022 [23] | 78 | 41 | NR | 78 | 21 | NR | 0.51 (0.31-0.88) |

SC-standard care; SD-standard deviation; CR-cardiac rehabilitation; HR-hazard ratio; NR-not reported
